# Adherence to Antipsychotics, COVID-19 and Stigma: A Rainbow After the Storm?

**DOI:** 10.62641/aep.v53i3.1813

**Published:** 2025-05-05

**Authors:** Luiz Dratcu, Xavier Boland

**Affiliations:** ^1^Acute Inpatient Psychiatry, Maudsley Hospital, South London and Maudsley NHS Foundation Trust, SE5 8AZ London, UK; ^2^Liaison Psychiatry Service, Lewisham Hospital, South London and Maudsley NHS Foundation Trust, SE5 8AZ London, UK

**Keywords:** antipsychotics, COVID-19, psychotic disorders, schizophrenia, stigma

## Abstract

Poor adherence to antipsychotic treatment is the main reason for relapse and admission in patients with severe mental illness (SMI). In addition to apprehensions about side effects, factors for non-adherence include the stigma of mental illness, which also extends to its treatment and which is further compounded by negative perceptions of antipsychotics within the medical profession itself. By reinforcing misconceptions around antipsychotics, warnings at the outbreak of pandemic associating antipsychotic use with coronavirus disease 2019 (COVID-19) related risks could have discouraged antipsychotic prescribing when it was otherwise indicated and also disrupted adherence of SMI patients to the treatment they need. Several studies on risk of antipsychotic use during the pandemic adopted inappropriate inclusion criteria and reached conclusions that proved misleading, particularly if applied to SMI patients. Methodological flaws included ill-defined cohort groups and not accounting for adherence to treatment or clinical indication for antipsychotic prescribing. Conversely, reports from the clinical setting, which were later corroborated in clinical studies, have shown that adherence to antipsychotics may actually protect from COVID-19, thus indicating that the benefits of adhering to antipsychotic treatment extend beyond controlling psychotic symptoms alone. Evidence emerging from the COVID-19 experience adds to the view that adherence to second generation antipsychotics in SMI offers a therapeutic effect to both mental and physical health. This validation of modern antipsychotics as a vital medical asset can prove a turning point in the fight to dispel the stigma of SMI and its treatment.

## Introduction: Adherence to Antipsychotic Treatment, Then and Now

Since the introduction of antipsychotics in the 1950s, mental health services have seen a radical transformation with the closure of asylums, reduction in the length of hospital admissions and the massive expansion of community services. Most of these changes are directly attributable to the success of antipsychotics in both the treatment and relapse prevention of severe mental illness (SMI). It has since been well established that antipsychotics are most effective in the preventing psychotic and manic relapses when taken regularly [1], yet non-adherence to treatment has persistently proved a major clinical challenge.

To this day, far below 50% of patients with schizophrenia [2] and bipolar disorder [3] are estimated to adhere to antipsychotic treatment. Whilst non-adherence to antipsychotics is the main driver of relapse and admission to hospital in SMI [2], the lowest rates of re-admission to hospital in patients with psychosis are in those prescribed long-acting second-generation antipsychotic injections, where adherence to treatment can be closely monitored by mental healthcare teams [4]. The coronavirus disease 2019 (COVID-19) pandemic has served as a stark reminder of how critical this issue remains to this date, both as a topic of research and in everyday clinical practice.

## To Adhere or Not to Adhere to Antipsychotics: Stigma and COVID-19

Multiple factors contribute to antipsychotic non-adherence in SMI, including disease-related factors (e.g., lack of illness awareness, psychiatric symptoms), patient-related factors (e.g., comorbid substance misuse, insufficient understanding of the disease and its treatment) and cultural and social factors [2, 5]. Among these, patients’ understanding of their illness and of the rationale for treatment seem to play a vital role in their willingness to adhere or not to treatment [6]. This should be seen within a wider context, involving not only patients but also prescribers, where culture-bound attitudes toward mental illness as a concept, and toward psychiatric treatments as evidence-based interventions, can also act as barriers to patients accessing antipsychotics when they are otherwise indicated [7].

The most glaring example of this is the stigma associated with mental illness, which predictably also extends to what many see as its proxy, namely antipsychotic treatment [8]. But this is a two-way street. While public- and self-stigma associated with the use of antipsychotics can indeed play a major part in patients’ decisions not to accept treatment [9], this cannot be isolated from psychiatrists’ own views on the treatments they are meant to offer their patients. After several decades of robust evidence demonstrating the therapeutic value of antipsychotics when properly prescribed, antagonism to using antipsychotics can still be found in scholarly publications both as a matter of principle [10], and thus impervious to evidence, and as a result of biased interpretation of it [11]. By reaching the public unchecked, antagonistic views of this nature can only reinforce existing negative or misinformed perceptions of antipsychotics, thus contributing to both perpetuate the stigma attached to antipsychotic treatment and undermine the scope for educating patients about the need to adhere to it.

Practising clinicians had good reason to fear that such an unvirtuous cycle of stigma around antipsychotic use could unfold during the COVID-19 pandemic. At the outbreak of the pandemic in early 2020, conflicting reports on the association between antipsychotics and adverse COVID-19 outcomes triggered a renewed debate on the safety of antipsychotics which could eventually expand to their place in the treatment of SMI itself. At a time of crisis, there might have been calls to discontinue antipsychotic treatment in patients who had been receiving it and a new reluctance to start antipsychotic treatment in those who needed it [12].

## Antipsychotic Prescribing and the COVID-19 Pandemic: Moving Posts 

Early in the COVID-19 pandemic, SMI patients were considered an at-risk group for poor outcomes given their higher rates of cardiometabolic disorders and smoking as compared with the general population [13, 14]. Urgent prescribing recommendations by expert consensus advised caution in the use of antipsychotic in SMI patients, highlighting the potential risk of antipsychotic-induced sedation increasing respiratory depression and also of thrombo-embolic events and secondary infections in COVID-19 [15]. Initial epidemiological studies argued that SMI patients were indeed more vulnerable to complications of COVID-19 by suggesting an association between antipsychotic exposure and hospitalisation and death from COVID-19 [16]. Whether or not these outcomes influenced antipsychotic use at that stage, some were quick to blame antipsychotics for COVID-19 complications in SMI regardless [11].

Later, a few clinical reports, alongside smaller studies addressing specifically the issue of antipsychotic prescribing and COVID-19 risk in SMI, challenged initial concerns about antipsychotic safety during the outbreak of the pandemic [12]. Then, scrutiny of earlier research based on electronic health records, which had raised warnings about potential medical risk associated with antipsychotic prescribing in COVID-19 patients, identified methodological shortcomings which undermined the validity of their findings [17]. Emerging evidence seemed increasingly to indicate that, on the contrary, antipsychotics were in fact likely to *protect* SMI patients from COVID-19 [18]. Firstly, by reducing the vulnerabilities to COVID-19 associated with untreated SMI, proper antipsychotic prescribing could help preserve patients’ physical health. Secondly, by dampening cytokine storms associated with complications of SARS-CoV-2 infection, the anti-inflammatory properties of some antipsychotics may also reduce adverse outcomes in COVID-19 [18].

## COVID-19 and Adherence to Antipsychotics: Clinical Outcomes 

Admonitions about antipsychotic use during the COVID-19 pandemic had proved premature. Of four major epidemiological studies based on large patient databases and which had found an association between antipsychotic prescribing and severe adverse COVID-19 outcomes, *all *included patients in their treatment cohorts who had only been *exposed* to antipsychotics but without reference to adherence to or indication for antipsychotics, nor to length of treatment [19, 20, 21, 22]. This is doubly problematic. Firstly, as up to 75% of antipsychotics prescribed in adults are done so on off-label indications and often in the absence of SMI, their results did not necessarily reflect the risk profile of SMI patients [23]. Secondly, their study outcomes would also not differentiate *treated* from *under-treated* SMI. Similar problems with inclusion criteria were also found in a prospective cohort study by D’Andrea *et al*. [24], which were acknowledged by the authors themselves.

Conversely, several studies subsequently demonstrated either no effect or a protective effect of antipsychotics against COVID-19 complications when accounting for adherence to treatment in their study design. For instance, a study by Canal-Rivero *et al*. [25], which looked specifically at SMI patients receiving long-acting injectable antipsychotics (where adherence can be closely monitored) and only included those with at least 80% compliance to treatment, found that patients with *treated *SMI had lower risk of COVID-19 infection and better COVID-19 outcomes than the population at large. A similar study by Ruiz de Pellón-Santamaría *et al*. [26], including only SMI patients receiving long-acting injectable antipsychotics, reported similar findings, even though it was underpowered.

Narrowing the scope of studies only to SMI patients admitted to psychiatric units during the pandemic also allowed to account for adherence to antipsychotic treatment, as this could be closely managed in the inpatient setting. Accordingly, studies by Prokopez *et al*. [27] and Nemani *et al*. [28] of inpatient psychiatric populations in Spain and the USA, respectively, both found that antipsychotics reduced the risk of COVID-19 infection. A later analysis of COVID-19 outcomes in SMI patients receiving antipsychotic treatment in New York state hospitals, also by Nemani *et al*. [29], found no association between antipsychotics and 60-day mortality from COVID-19 (odds ratio, 1.00; 95% confidence interval 0.48–2.08; *p* = 0.99). Of relevance, authors excluded patients with antipsychotic discontinuation or non-adherence from the exposure group. It thus seems that an overreliance on epidemiological research derived from large medical record databases, at the expense of clinical observations or experimental and clinical research, can limit the ability to answer complex clinical questions, such as those associated with the needs of SMI patients [12].

## Antipsychotics: More Than Controlling Psychotic Symptoms Alone

Antipsychotics are not perfect and are not all the same, nor are they always used as they should be [12]. It is obviously incumbent on clinicians to prescribe safely and effectively to patients under their care. On this note, the experience of treating SMI patients during the COVID-19 pandemic has offered practising psychiatrists some valuable lessons of direct impact on patient care. First, the pandemic has shown that research on antipsychotic use in SMI which fails to consider adherence to treatment can produce results which are unrepresentative of both clinical outcomes and patients’ experience in the presence of good prescribing practices. Evidence arising from the COVID-19 pandemic further reinforces not only the benefits of prescribing antipsychotics when they are indicated but also the need of supporting SMI patients to remain on treatment. Accordingly, clinicians should be aware of and assess critically the inclusion criteria and methodology of database analyses that claim otherwise [17].

Second, the potentially protective effect of antipsychotics against COVID-19 in SMI seems to add to other medical benefits that adherence to treatment may bring to this patient group. For example, patients with schizophrenia have on average over fourteen years reduction in life expectancy as compared with the general population [30]. And yet, despite the cardiometabolic effects associated with some antipsychotics, antipsychotic treatment has been shown to be associated with a dramatic reduction in mortality in schizophrenia patients. A 20-year follow-up study of 62,250 patients with schizophrenia in Finland by Taipale *et al*. [31] used national prescription register data to compare patients with >80% adherence to antipsychotic treatment with those who either had full (0%) or partial non-adherence (<80%) to treatment during outpatient observation time. The study found cumulative mortality rates of 46.2% in the non-adherence cohort and only 25.7% in patients who were adherent to antipsychotic treatment. Similarly, a Swedish study by the same group, of 29,823 patients with schizophrenia followed up over a period of 5.7 years, also found a further 33% reduction in mortality in patients on long-acting injectable antipsychotics as compared with those on oral antipsychotic formulations [32].

More broadly, adherence to antipsychotics in schizophrenia contributes to a reduction in healthcare utilisation and costs [33] and has been associated with a reduction both in use of emergency services [34] and discontinuation of drugs prescribed for the treatment and prevention of cardiovascular disease [35]. Further research is clearly required to further explore the effects of evidence-based antipsychotic treatment on the physical health of SMI patients, particularly in the long-term. Nonetheless, if recent findings are any guide, the therapeutic effects of antipsychotic use in SMI are likely to be increasingly understood as extending beyond controlling psychotic symptoms alone.

## Reframing Contemporary Antipsychotic Treatment

Finally, after the tragedy of the pandemic, a positive and enduring legacy of the COVID-19 experience for mental healthcare may lie in the unique opportunity it offers to radically change the public perception of antipsychotic treatment. For one, at a critical time and under unprecedented circumstances, it has again been shown that, when properly prescribed, antipsychotics are a safe and effective treatment that can counter the many devastating consequences of SMI. Beyond that, the finding that adherence to antipsychotic treatment may protect SMI patients against COVID-19 resonates with the mounting evidence indicating it can also protect them from the medical comorbidity from which they suffer. This brings a renewed emphasis to the notion that patients’ mental health is not separate from their physical health, and a compelling message to SMI patients in particular, but also to the public at large, that adherence to antipsychotic treatment is beneficial on both counts, to the extent it can potentially contribute to prolong patients’ life expectancy [30, 31].

As such, antipsychotics should be seen as yet another life-changing breakthrough that modern medicine can deliver in response to the litany of disabling clinical conditions which were for ages beyond the reach of any treatment, among which those classed as SMI stand out as a prominent example. Accordingly, therapeutic innovations in mental healthcare, like long-acting second-generation antipsychotics, should henceforth be welcomed as an invaluable medical resource that, as well as treating psychotic symptoms, can change SMI patients’ lives for the better [4].

## Conclusion

By challenging negative misperceptions of antipsychotic treatment, a freshly invigorated post-COVID-19 view of the choice of currently available antipsychotics as a fully evidence-based asset of modern medicine is bound to contribute to dispelling the cycle of stigma associated with SMI and its treatment. Perhaps unexpectedly, the COVID-19 experience may thus have brought practising psychiatrists a broadened scope for educating SMI patients about antipsychotic medication and a renewed impetus for actively engaging them with the treatment they need. This should inspire clinicians to develop newer and creative partnership strategies in supporting SMI patients to adhere to their treatment, now incorporating long-acting second-generation antipsychotics as integral part of their medical healthcare.

## Availability of Data and Materials

Not applicable.
